# Length of service and commitment of nurses in hospitalsof Social Security Organization (SSO) in Tehran

**Published:** 2014

**Authors:** Seyed Ebrahim Jafari Kelarijani, Ali Reza Heidarian, Reza Jamshidi, Mohamad Khorshidi

**Affiliations:** 1 Treatment Management of Social Security Organization of Mazandaran Province, Ghaemshahr, Iran.

**Keywords:** Nurse, Organizational Commitments, Length of Service, Hospital

## Abstract

***Background: ***A nurse’s commitment is the most important factor that influences her performance and depends on other variables. The purpose of this research was to study the relationship between length of service of the nurses with the amount of occupational commitment and organizational commitment.

***Methods:*** From Winter 2012 to Spring 2013, 266 nurses were chosen in selected hospitals of Social Security Organization (SSO). These nurses were randomly categorized into six different classes of service records including < 5, 5-9, 10-14, 15-19, 20-24, and 25-29 years. The length of service is related to the organizational, occupational, affective, continuance, and normative commitment. The data were collected and analyzed.

***Results:*** Generally 84% of the responders were women and the rest were men of which 95% had a bachelor’s degree and the rest had higher academic degrees. The length of service in 81% of nurses was <15 years and 19% were higher than 15 years. Significant correlation were seen between continuance and occupational commitments and length of service (r=0.23, P=0.04 and r=-0.26, P=0.02, respectively). There were not any significant differences regarding organizational, affective and normative commitments (P=0.12, P=0.33, P=0.47, respectively).

***Conclusion: ***The results show that the length of service was related to continuance and occupational commitment. So pre-retirement of the nurses after 20 years of work can result in an increase in average commitment of employees.

Organizational success depends heavily on employees, as they are perhaps the only source of sustainable competitive advantage to organizations (1). Understanding the process that leads to the increase of commitment in an organization is critical for building stability and job security and increase of organizational effectiveness. Regarding the role of nurses especially in hospitals, there is a strong need to increase their commitment in institutions that provide health services to patients (2). Commitment has been studied extensively in terms of its components, antecedents, correlates, and consequences and with the development of the concept of commitment, an associated critical literature has arisen (3-9). A study done by Chang and Choi in 2007 was designed to determine the effect of years of service on the organizational commitment of workers (10). The purpose of this research was to study the relationship between the length of service of nurses with different commitments. 

## Methods

This study was conducted from Winter of 2012 to Spring of 2013 in selected hospitals of Social Security Organization (SSO) in Tehran. The research sample consisted of 266 nurses categorized into six different classes of service records, less than 5, 5-9, 10-14, 15-19, 20-24 and 25-29 years who were selected randomly. The present study employed a questionnaire survey approach to collect data for testing the research hypotheses. The variables that were used in this study are length of service and occupational commitment, organizational commitment, affective commitment, normative commitment and continuance commitment. These variables were marked based on a five-point Likert style level of response ranging from “strongly disagree” to “strongly agree”. (1. Strongly disagree, 2. Disagree, 3. Neither agree nor disagree, 4. Agree, 5. Strongly agree). 

**Commitment: **Commitment is the strength of an individual’s identification and involvement in a particular organization and it is a construct that seeks to explain consistencies involving attitudes, beliefs and behavior and “involves behavioral choices and implies a rejection of feasible alternative courses of action. Thus, these consistencies are usually seen as behavioral choices devoted to the pursuit of a common goal or goals (11, 12). 

**Occupational commitment: **Occupational commitment is the relative strength of identification and involvement in a particular profession, as well as the willingness to exert effort on behalf of the profession and the desire to maintain membership in it (13). 

**Organization commitment: **Organizational commitment is regarded as a mental contract connecting the individual’s identification and attribution with the organization and performing his duty (14). Employees’ commitment in the organization can take various forms, and the antecedents and consequences of each form can be quite different (5-6). We also identified and developed measures of three major components of organizational commitment: affective, continuance, and normative (6). Affective commitment reflects an emotional attachment to identification, and involvement in the organization. Continuance commitment is based on the perceived costs associated with discontinuing employment with the organization. Finally, normative commitment reflects a sense of obligation on the part of the employee to maintain membership in the organization (6).

**Statistical analysis: **Reliability was calculated via Cronbach's Alpha (r=0.84) and data analysis was done by SPSS Version 13. Correlation between variables to analyze the organizational commitment, occupational commitment and length of service, Spearman Correlation coefficient was used. Spearman test was used to evaluate the correlation between the quantitative and qualitative variables. 

## Results

Demographic results of the study show that 84% of sample population were females and 16% males. From the education, 95% had bachelor’s degree and the rest had higher academic education. Also, 18% of sample population was between 0 and 4 years length of service, 35% between 5 and 9, 28% between 10 and 14, 10% between 15 and 19, 5% between 20 and 24, 4% between 25-29. Actually 82% of them were between 25-40 years old and the others over 40 years old. Table 1 shows the mean and standard deviation of all variables. Table 2 presents the results of correlation analysis regarding the effects of length of service on organizational commitment and occupational commitment. 

**Table 1:** Means of organizational commitment and dimensions and occupational commitment of nurses in selected hospitals of Social Security Organization in Tehran

**Length of Service**	**Variable**				
	**Organizational** **Commitment** **Mean** **±SD**	**Affective** **Commitment** **Mean±SD**	**Continuance** **Commitment** **Mean±SD**	**Normative** **Commitment** **Mean±SD**	**Occupational** **Commitment** **Mean±SD**
0- 4	4.33±0.07	4.54±0.07	4.04±0.07	4.4±0.07	4.17±0.23
5- 9	4.19±0.07	4.43±0.08	3.96±0.07	4.18±0.07	4.25±0.23
10-14	4.00±0.07	4.2±0.12	3.8±0.44	3.9±0.12	4.14±0.2
15-19	4.21±0.07	4.8±0.2	3.8±0.42	4.02±0.13	4.25±0.32
20-24	4.1±0.27	4.2±0.22	4.1±0.25	4.2±0.22	3.8±0.16
25-29	4.7±0.13	5.1±0.13	4.4±0.13	4.6±0.16	3.5±0.18

**Table 2:** Relationship between length of service and organizational commitment and dimensions and occupational commitment of nurses in selected hospitals of Social Security Organization in Tehran

	**Variable**				
	**Organizational** **Commitment**	**Affective** **Commitment**	**Continuance** **Commitment**	**Normative** **Commitment**	**Occupational** **Commitment**
Length of Service	r= -0.05 P= 0.12	r= -0.17 P= 0.33	r= 0.23 P= 0.04	r= 0.12 P= 0.47	r= -0.26 P= 0.02

The following results have been revealed according to findings considering the main object of the project: generally, the mean score of organizational commitment was (4.17±0.08). Affective commitment had the highest amount (4.44±0.11) and continuance commitment the least (3.94±0.22), normative commitment was (4.14±0.1), and occupational commitment (4.15±0.23) (table 1). 

There was not a relationship between the length of service and organizational commitment, normative and affective commitment according to *correlation* statistical analysis test (P=0.12, P=0.47, P=0.33, respectively). But there was a meaningful relationship between continuance and occupational commitment and length of service (P=0.04, P=0.02) (table 2).

## Discussion

Employees’ commitment to an organization is necessary for contemporary organizational success because it positively affects job performance (15-16). Investigation about commitment is more important for managers and organizations (1, 17). Hence, it is important to understand how organizational commitment and occupational commitment affect contemporary employees, especially the professionals who choose to leave their organizations for professional advancement (18).

This study examined the role of length of service on organizational commitment and occupational commitment. Our results indicate that length of service has significant effect on continuance commitment and occupational commitment. These findings highlight the critical roles of years of service in continuance commitment of the nurse's population in hospitals. With increasing years of service in nurse's society, the occupational commitment decreased and continuance commitment increased. In this study, the length of service was positively related to organizational commitment (general), affective commitment and normative commitment of the nurses. The results of the correlation analysis indicated that there is not a relationship between the length of service and organizational commitment (general), affective and normative commitment. Popoola found that the personal factors like length of service affect the organizational commitment of records management personnel in Nigerian state universities (19). In the same vein, studies indicated that organizational commitment followed a U-shaped pattern, while occupational commitment followed the inverse of the U-shaped pattern over the same period of years of services (10).

More studies similar to our own, McNeese-Smith and Mowdy et.al, analyzed the effects of years of service and commitment found that both the organizational commitment and occupational commitment have a strong positive correlation with years of service in employees (20, 21). These studies have similar results in this paper about occupational commitment and continuance commitment and in conflict with current research about organizational, commitment, affective commitment and normative commitment in which these differences may be searched in the nature of the commitment component. However, Steers reported that there was not any significant correlation between age, length of service and organizational commitment (9) wherein this finding also confirms one of the hypothesis about the relationship between length of service and organizational commitment (general).

In conclusion, the results show that the length of service was related to continuance commitment and occupational commitment. There were no relation between other variables. Also, pre-retirement of the nurses after 20 years of work can result in an increase in average commitment of the nurses. The possible limitations were the effect of social desirability that existed on the part of employees. Because the respondents were asked to answer questions about their feelings toward their job and organization, it was possible that they might have answered the questions according to the expectations of others. Employees may have felt hesitant to respond honestly to the survey because of fear that their information would be disclosed. Another limitation of this study was the nature of the sample. The participants were selected from hospitals in Tehran. Therefore, the generalization cannot be made to the entire population of other similar hospitals. Future studies can also examine the proposed relationships in other countries.

In summary, the results show that the length of service was related to continuance commitment and occupational commitment. There were no relations between other variables. Also, pre-retirement of the nurses after 20 years of work can result in an increase in average commitment of the employees.

## Notes

**Funding:** This study was supported by a research grant from the Social Security Organization in Tehran.
